# Understanding interpretations of credibility, health literacy and health information seeking in Black and South Asian ethnic communities in the United Kingdom: A qualitative study

**DOI:** 10.1371/journal.pone.0355294

**Published:** 2026-09-02

**Authors:** Tushna Vandrevala, Kristin Hanson, Celayne Heaton-Shrestha, Richard Boulton, Lindsay M. Bearne

**Affiliations:** 1 Faculty of Health, Science, Social Care and Education, School of Nursing, Allied and Public Health, Kingston University, London, United Kingdom; 2 Department of Psychology, Kingston University, London, United Kingdom; 3 Population Health Research Institute, City St George’s, University of London, London, United Kingdom; Taylor’s University, INDONESIA

## Abstract

Health literacy is essential for making informed healthcare decisions, yet ethnic minority communities often face disparities due to limited access to culturally relevant information. This qualitative study explores how South Asian and Black communities in the United Kingdom (UK) assess health information credibility, examining cultural influences and expectations. Sixty-eight participants (42 South Asian, 26 Black) were recruited using purposive sampling; data collection involved semi-structured interviews (n = 30) and community observations (n = 38). Findings revealed that cultural expectations shape perceptions of healthcare professionals and health information sources. Trusted sources included personal contacts and social media. Health information lacking personal or culturally relevant content led participants to seek alternative sources. While some credibility-seeking behaviours overlap with those observed in the general population, the unique social, cultural, and historical experiences of these populations shape how and why such behaviours are enacted, resulting in distinct patterns of trust and information use. Providers must recognise these dynamics to create personalised, culturally sensitive resources that enhance trust, health literacy, and equity. Tailored approaches, including trusted intermediaries and relevant communication channels, are crucial, while digital platforms must address misinformation.

## 1. Background

Health and social care systems rely on health literate individuals who possess the knowledge and digital skills to navigate, access, process, and comprehend health information, that enables them to make informed decisions about their healthcare [1]. Individuals with limited health literacy may struggle to obtain or understand crucial health information related to prevention, illness, and treatments [2,3]. Furthermore, people with limited health literacy are more likely to use and trust sources that are associated with ‘misinformation’—including social media and friends—and less likely to use official medical websites [4]. Low health literacy levels reflect broader societal inequalities and are more prevalent in, though not unique to, ethnic minority groups [5]. Recent studies, that have explored perceptions of health information sources, primarily focus on specific websites or conditions within the general population [6,7]. An extensive review of the literature was not able to locate studies pertaining specifically to assessments of credibility in minority populations [6].

### 1.1. Health literacy

The concept of health literacy has been widely employed over the past three decades to measure and explain health inequalities, with lower health literacy associated with decreased participation in preventive health services and poorer overall health status [5]. Most studies on health literacy have focused on an individual’s ability to acquire, process, and use information, but has paid little attention to the interface between members of the public, communities, healthcare providers, and healthcare systems [8]. By ‘community’, we mean a group whose members engage in regular interaction, have shared interests, and proximity in geographical or virtual space [9].

The increasing emphasis on health information-seeking and an individual-centric approach to health literacy may inadvertently exacerbate health disparities – those who can easily access health information have an advantage over individuals who encounter challenges in accessing information. Given that health literacy tends to be lower among ethnic minority groups compared to the general population, these groups encounter barriers related to accessing health information, including language barriers, cultural relevance of information, ability to comprehend material and high rates of digital exclusion [10]. Without improvements in health literacy and the accessibility of health information for ethnic minority groups, health disparities are likely to escalate.

In recent years, there has been a shift in how health literacy is understood, moving from an individual-focused perspective to a broader societal and organisational perspective. The United States (US) Department of Health and Human Services acknowledged this change by stating that health literacy is achieved when accurate health information and services are readily available and easily understood by individuals [11]. This shift in the discourse emphasises the role of organisations, professionals, and systems in creating and delivering health information that enables individuals and communities to access, comprehend, and use health information and services for informed decision-making and actions. Research has emphasised that there is a mismatch between the complexity of health information provided by authorities and the literacy skills of the general population and a need to increase the simplification of, and community representation in, health communications [12]. However, these conceptualisations of health literacy are incomplete as they do not account for the health information source preferences and credibility of health information that may vary in members of ethnic minority groups.

In the UK, General Practitioners (GPs) and the NHS website are widely recognised as primary sources of mainstream health information. GPs serve as the first point of contact for most health concerns and are central to the delivery of primary care services. The NHS website, maintained by the National Health Service, provides official guidance on symptoms, treatments, and service access, and is often promoted as a reliable and authoritative source. These sources are typically positioned as the default or expected channels for health information within UK health systems and health messaging. Mainstream health information often reflects the norms and expectations of dominant cultural groups, which may make it more accessible to those populations. However, this same framing can inadvertently exclude ethnic minority communities, whose health information needs, cultural practices, and credibility assessments differ in important ways.

Members of ethnic minority groups may differ from the general population in their assessment of health information credibility. This might be influenced by their lived experiences, cultural influences, and experiences with healthcare systems. For immigrants, this may include systems in both their country of origin and their host country. Trust in institutions and service providers may also be lower in marginalised communities compared to the general population, often attributed to past experiences of prejudice [13]. A difference in the assessment of trust, as a collective characteristic, can substantially impact the evaluation of health information sources used and health-seeking behaviour [14,15].

Additionally, cultural influences, comprising sets of beliefs and behaviours, shape individuals’ perception, comprehension, and evaluation of health information credibility [16]. Social and religious beliefs regarding health interventions can affect individuals’ evaluation of health information sources, messages, and channels [17]. Evidence suggests that, in minority communities, both medical expertise [18] and lay opinions [19] are considered when assessing the credibility of information sources. For some ethnic communities, family and community play crucial roles [20] in this assessment.

### 1.2. Assessment of health information credibility

One way to understand health information source preferences in ethnic minority communities is to investigate perceptions of source *credibility*. Hovland et al. [21] defined credibility as consisting of perceptions of information source *trustworthiness* and source *expertise*, where trustworthiness refers to ‘the degree of confidence in the communicator’s intent to communicate the assertions he considers most valid’, and expertise means ‘the extent to which a communicator is perceived to be a source of valid assertions’ [21]. Expertise may be based on credentials such as medical qualifications or other criteria, including lived experience [22].

The current study aimed to investigate how people from two ethnic minority (Black and South Asian) communities living in the United Kingdom (UK) perceive health information credibility. This study aimed to consider how cultural influences and expectations affect health literacy, and shed light on ways to enhance information channels, sources, and formats to encourage health information seeking and health literacy, within these communities. Understanding the assessment of health information credibility within ethnic minority groups will help health information providers know how to best impart health information.

## 2. Method

### 2.1. Study design

The study used a qualitative approach employing semi-structured interviews and observations as they allow for an in-depth, culturally-nuanced exploration of community perspectives and experiences, providing insights into unique information needs and barriers to access encountered by these communities [23]. We also valued the ability of qualitative methods to foster collaborative and participatory research [24]. Ethnically diverse members of the Community Advisory Group (CAG) helped develop our approach and methods. This was considered important given that participatory methodologies are best practice when involving underserved and vulnerable groups in research [25].

All procedures were performed in compliance with relevant laws and institutional guidelines and have been approved by Kingston University Research Ethics Committee (Ref: 2977) on ^th^e 4^th^ Feb 2022. The privacy rights of human subjects have been observed and that written informed consent was obtained for experimentation with human subjects.

### 2.2. Participant sample and recruitment strategy

The study recruited participants identifying as South Asian (Indian, Pakistani, Bangladeshi) and Black (African, Caribbean), communities constituting the two largest ethnic minority communities in the UK (respectively, 7% and 3.5% of the total population) [26]. Sampling was purposive [27] in keeping with the objectives of the study, and to ensure richness of information from participants knowledgeable on the topic. Criteria for inclusion in the study were: self-reported membership of target ethnic group, age over 18 years, willingness to participate in the study and ability to give informed consent. Participant information sheets were provided to prospective interviewees who expressed interest in the study to researchers, and to the leaders of the community settings, who provided the information to community members.

Potential participants were recruited into one of two phases to ensure that we could recruit participants with differing levels of health literacy and trust in research. In Phase 1 semi structured interviews included participants with high literacy while phase 2 included those with lower literacy and used observations to collect data and generate more engagement -the details of which are in the next two sections.

#### 2.2.1. Phase 1: Interviews.

Interview participants were recruited through social media platforms, the research team’s links to community networks (e.g., through past projects with voluntary community and social enterprise organisations), and through the study’s Community Advisory Group (CAG), comprising a patient advisor, an asylum seeker, and leaders of charities serving communities from lower socio-economic backgrounds. Prospective interviewees were encouraged to contact the researchers directly to arrange an interview. All interviews were conducted in English by a White female researcher (KH), employed as a research fellow at the university, with extensive experience and training in social science research and holding a PhD in psychology.

As data collection progressed, and consistent with the iterative nature of purposive sampling, where researchers recruit additional participants as understanding develops (Schutt 2006), we included a second group of participants (phase 2 participants) with different health literacy and educational levels from the first group of participants, to ensure heterogeneity within our study sample.

#### 2.2.2. Phase 2: Observations of participants from community events.

To diversify our sample, two researchers (TV, CH-S) attended two community events facilitated by the leader of the charity and four Health Advocates from the community. These female researchers have a background in psychology and social anthropology and hold PhDs. One of the researchers also identifies as South Asian, and the other as White. Observations in the community settings provided access to participants with more limited English, recent migrants, persons residing in deprived neighbourhoods, and those less likely to come forward for interviews. The community events were run by a charity that focuses on empowering socially disadvantaged ethnic minority communities. The charity holds weekly community-based events to discuss the community’s unmet health and wellbeing needs, and also to address community cohesion and social isolation. The local charity is well known and respected by the community in London (Westminster Borough) and has operated for nearly 20 years. The leader repeated and clarified the purpose of our visit to participants at the beginning of the observed events.

#### 2.2.3. Consent of participants.

Participants in phase 1 (interviews) were asked to complete a consent form. Participants in phase 2 (observations) were informed of the purpose of the meeting ahead of time, and offered the option to not take part, and those attending the event were informed at the outset that they did not have to say anything if they did not wish to, with no adverse consequences on their future participation in charity activities. Both prospective interviewees and community setting participants were afforded the opportunity to ask questions about the study

### 2.3. Tool development and data collection

A semi-structured interview schedule (see Appendix 1) was developed with members of the Community Advisory Group (CAG) and piloted with two members from the target ethnic communities. The interview schedule aimed to explore participants’ health information-seeking preferences and included questions such as ‘Where do you go for health information?’ and ‘How do you decide to trust the information you access?’ Questions and prompts were designed to encourage participants to share their personal experiences and perspectives on how their family or community members access and evaluate health information.

Interviews were conducted online ranging from 20 to 60 minutes in duration and either video or audio-recorded,. Four participants chose to turn off their cameras during the interview. The interviews were conducted by one researcher (KH) with debriefing opportunities and support from (TV) during February and March 2022.

The observations in community settings took place during two 2-hour in-person visits in February and March 2022. The method of data collection (observation of exchanges and informal conversation with participants on the topic of interest, rather than individual interviews) was largely dictated by the charity leader who facilitated access to these settings. The discussions followed a schedule of guiding questions based on the interview schedule and were facilitated by the charity leader and the health advocates. Two researchers (TV, CH-S) attended the community event as observers and asked questions and sought clarifications during the discussions when required. After the formal event, participants approached the observers to engage in further discussions on relevant topics. At the request of the charity leader, the events were not audio-recorded to ensure anonymity and to maximise participation. The two researchers made verbatim written notes of exchanges between participants on the topic of interest. Notes taken during the event were expanded, and the two sets of notes were subsequently cross-checked by the two researchers attending the event.

### 2.4. Data analysis

Interview data was transcribed verbatim and then uploaded to MAXQDA2020, a qualitative data analysis software package, to facilitate analysis. To ensure participant anonymity, pseudonyms were used throughout the study. Reflexive thematic analysis was employed to identify and analyse the themes within the data, utilising both inductive and deductive coding techniques, following Braun and Clark [28]. The initial data analysis was performed by (KH), who familiarised themselves with the entire data corpus before proceeding with coding. Using an inductive approach, an initial thematic map was generated. The analysis process involved a deductive review of the data to identify any passages that pertained to the concepts of trust, expertise, or credibility. Codes were refined and consolidated through consultations and discussions between four team members (TV, KH, CH-S, LB). This iterative process aimed to ensure the comprehensive exploration of relevant themes and their connections within the data. The comprehensive notes from the observational visits were also subjected to thematic analysis**.** Braun & Clarke [29] argue that Constant Comparative Analysis, specifically associated with Grounded Theory approaches, is foundational to all good qualitative practice. The material collected from observations was reflected against the data collected from interviews, through systematic comparison of the two bodies of qualitative data. Data saturation was discussed within the team and though some areas needed further exploration (such as the role of religious framing and discussions about health and the lack of diversity among Black participants), the decision was made to explore this in future studies.

To ensure the quality of our study and its findings, we followed the four criteria of trustworthiness of qualitative research developed by Lincoln and Guba [30]. Specifically, we sought to establish credibility through peer debriefings to check interpretations (during meetings with the CAG, and with the charity leader and health advocates). Research team member (TV)’s long-term engagement with the target communities and charity further supported the credibility of the study. The transferability criterion was met through providing sufficient detail of the participants and including verbatim quotations from participants. To ensure the dependability and consistency of our study, we created and maintained an audit trail, consisting of full verbatim interview transcripts and notes of observations.

## 3. Results

### 3.1. Participant characteristics

The mean age of the 30 interviewees (Phase 1) was 38 (range 23–66) years old. Participants included: 16 who identified as male, and 14 as female; 16 who identified with a South Asian ethnicity, and 14, with a Black ethnicity; 13 were born in the UK, and 17 were born outside of the UK. 13 participants resided in London, four in the South East, five in the North West, one in the North East, two in the West Midlands, two in the East of England, two in the South West and one in Scotland. All interviewees had high health literacy levels, as most had high educational attainments, with 13 having studied at Undergraduate level and 11 at Postgraduate level, with only six participants who had below undergraduate education.

A total of 38 participants were included in the phase 2. All participants were female, aged between 35–75 years, 26 identified as South Asian and 12 as Black and Afro-Caribbean. Fluency in the English language varied, but Health Advocates translated from the participants’ languages to English (and conversely), where required. No participants dropped out of the study.

Three major themes were developed from the analysis of the material, as presented below.

### 3.2. Assessments of health care providers as sources of health information: Influence of past experiences and cultural expectations

The first theme concerns the ways in which trust and expertise assessments of sources of health information were found to be strongly influenced by cultural expectations of, and past experiences with healthcare providers (HCPs), and how adverse experiences of self or community members led to the search for non-mainstream sources of health information. By ‘cultural expectation’, we mean a learnt and shared sense of what can (or cannot) be expected in relation to healthcare providers [31]. An important aspect of this theme was the stark difference between the experiences and expectations of participants belonging to the two target ethnicities. Both communities valued HCPs as sources of health information but differed in relation to the reasons for not drawing on this as a preferred source of health information. In one community, this was due to unmet expectations of their General Practitioners (GP) and, in the other, realised or feared expectation of poor or discriminatory treatment by HCPs.

#### 3.2.1. A highly revered personal contact and accessible source of health information.

HCPs, and notably the local GP, were considered a major and valued source of health information by many participants who were interviewed and observed in community settings. This was salient in the narratives of participants of older and first-generation migrants of South Asian ethnicity. One interviewee, for example, explained:

*They are the first point of contact. If anything, call GP…. If the GP give you first- hand information, I don’t need to go to third hand for information, because it’s not…reliable… He’s a doctor. He’s my GP. (Interview participant 10, SA male, 49)*

For these participants, the GP represented both a trusted personal connection and a source of expertise. Interviewees commented that in their countries of origin, they had had access to a GP who, as well as possessing extensive knowledge, was intimately familiar with their families and medical histories, and could provide them with health information and emotional support, tailored to their personal and family circumstances and appropriate for cultural norms, as needed. Some interviewees emphasised these GPs’ exceptional abilities, observing that medical practitioners were revered and often described in religious terms:

*In Asian culture, medical professionals are considered as a part of Jesus. …To describe a doctor or nurse or health professional, they will use the word, ‘Messiah.’ (Interview participant 30, SA male, 66)*

*No one in our community is more sacred than the GP. (Community setting participant, SA female)*

#### 3.2.2. Accessibility of GP – a source of frustration.

In contrast, GPs in the UK were perceived by participants to be less accessible than in participants’ country of origin, and this was a source of frustration.

*Back in India….everyone has a family doctor, so anything happens, you call. And they don’t mind taking your call, even if it’s 11pm … In UK, you don’t get that emotional support from your doctor. (Interview participant 2, SA female, 48)*

Other interviewees reported they found the process of scheduling appointments, seeing different healthcare providers who were unfamiliar with their medical backgrounds, and a lack of emphasis on addressing emotional needs in the UK, frustrating. GPs were perceived as unwilling to give time to their patients:

*Doctors don’t have time to sit with you… after 2 minutes ‘ok you go, that’s it’. (Community setting participant, SA female)*

Therefore, the GP in the UK was perceived as challenging to access and had little local or familial knowledge, in comparison to their health provider back in their countries of origin, although still trusted. Being directed by a GP to a website for further information on their condition left participants feeling that their concerns had not been taken seriously.

*I had a pain in the side and called the Doctor, I could not get an appointment. Then they told me to look at a video and go to A & E. I didn’t go to A & E. I called GP again, they gave me something to take. Then I told [the charity leader, who wrote a note to the GP and sent the participant to hospital]. They took an X-Ray, the bone was broken. It took three months. (Community setting participant, SA female)*

Consequently, some participants turned to other sources and channels for health information. One participant explained how they had turned to family in another country for information after feeling dismissed by their GP.

#### 3.2.3. A more ambivalent relationship.

The preference for a GP was also widely expressed among participants of Black ethnicity. They too, in common with the participants from the South Asian community, found the NHS website that they were directed to an inadequate replacement for in-person interaction with their GP and for receiving emotional support.

*Why should we go to the NHS website? Why is there no other option? I want a person. (Community setting participant, Black female)*

However, while expressing a preference for ‘a person’, members of the Black community perceived GPs less positively than South Asian participants.

*The medical professionals are like ‘drug pushers’. When you go to them, they just go [participant scribbles on a piece of paper]: ‘Here, take that’ (Community setting participant, Black female)*

This view was reflected in Black participants’ ranking of GPs as a second port of call for health information:

*You try your own remedy first, and if it fails, you go to the GP. (Community setting participant, Black female)*

*If I am showing symptoms, I will probably put the symptoms into Google…[or] the NHS website…[T]hat might then lead to my GP. But that usually comes further down. (Interview participant 26, Black male, 31)*

Discussion within the community events also highlighted gender differences in health information preferences, and the intersection of ethnicity and gender in shaping preferences. Female participants in the community event explained that, while they sought health information from GPs (albeit as a second resort), their male relations from their Black communities tended to avoid GPs because they feared stigma and discrimination:

*The men in our community…the doctor is seldom like them; and there’s a stigma, ‘oh, he’s got a mental health problem’ or the doctor is starting to demean them…People don’t have a good outlook on men, on a young black boy.* (*Community setting participant, Black female)*

From this exploration of participants’ preferred source of valid, trustworthy health information, we identified that there was a strong preference for HCPs (especially GPs) as a source of health information among participants of South Asian ethnicity as well as high credibility (i.e., perceptions of this source as trustworthy and expert). While GPs were mentioned as a valuable source among Black ethnicity participants, they were not always trusted because participants were not confident that they would be treated with respect (see earlier ‘drug pusher’ quotation). We also identified that in both instances, past experiences and expectations played a role in these assessments of this source of health information, and also shaped health information seeking behaviour.

### 3.3. Reliance and trust in personal social networks in-person and online

The second theme speaks to the value, trust and reliance on community sources of health information that are accessible through an individual’s personal social networks and community settings – including friends, family, religious and community leaders, barbers, shopkeepers with herbal knowledge, and setting such as clubs, adult education classes or workshops, and religious establishments (church or mosque).

#### 3.3.1. Accessing health information from family and friends.

Participants from the Black and South Asian communities emphasised the significance of personal connections, such as friends and family, in their pursuit of health information. This was salient in discourses from participants of South Asian backgrounds, though a smaller number of interviewees of Black ethnicity also indicated family or family members trained as HCPs as a particularly important source of health information.

*I don’t know many South-Asians who won’t have a family member who’s not working in healthcare. So we tend to, generally, connect with people in our community who works in healthcare.” (Interview participant 9, SA male, 40)*

*My friend, she is a psychologist…she specialises in mental health … I do have a quite a rich resource of people. (Interview participant 26, Black male, 31)*

Participants described a mutually beneficial relationship between the younger and older generations. The younger sought the life experience, knowledge and expertise of elders in relation to, for example, herbal and home remedies, while elders sought assistance with accessing health information and navigating the healthcare system:

*Sometimes I get advice from family members … [on] inherited diseases…that they may have experienced... Sometimes natural methods to…alleviate a certain issue. (Interview participant 6, Black female, 35)*

*I’m okay with my English… but my dad isn’t, so he would rely on us to be some sort of information giver. (Interview participant 11, SA female, 28)*

Community event participants predominantly relied on their families to contact the GP on their behalf and, in certain instances, to make healthcare decisions for them, for example:

*[Participant X] says that she has her husband and son to call the GP. Family members speak on her behalf... Sometimes members of the family make the decision. (translation by health advocate, Community setting participant, SA female)*

Interviewees and community event participants also highlighted the importance of community members other than family to provide expertise.

*People are just exchanging information from each other, from all of us Eritreans, the community. For example, if someone has any health issue, he calls his friend. He is asking, has it happened, this kind of stuff. Have you experienced this kind of thing? (Interview participant 27, Black male, 47)*

#### 3.3.2. Value in seeking health information via wider personal contacts in diaspora and country of origin – online and in person.

Some participants placed a particular value on health advice that aligned with the beliefs of community leaders, including religious leaders in their search for relevant and perceived expertise in health information:

*For example an imam, if he’s advocating that it’s absolutely fine to have your vaccination, …then that … just gave me some reassurance and my family that actually, yes it must be okay. (Interview participant 14, SA male, 51)*

Participants listed further venues, such as luncheon clubs, English language classes and places of worship that they would use to access information relevant to their health:

*So, we have to see, where do they meet. They go to the mosque, men, and women they have their own luncheon clubs, men have their luncheon clubs, knitting classes, ESOL [English to Speakers of Other Languages] classes. (Interview participant 30, SA male, 66)*

*My son went to the barbers’ and came back – I had never heard so much [health-related] information from his mouth! (Community setting participant, Black female)*

Information about natural remedies was accessed via shopkeepers from their communities:

*West Indian people do not go for conventional medicine, they are more into herbs… West Indian shops sell leaves, and they have information… they [shop keepers] will tell you what a leaf does. (Community setting participant, Black female)*

Social media played an important role in accessing health information as it supported the widely expressed preference for, and trust in, personal recommendation and personal contact – whether the individual contacted through these media was personally known or not (e.g., only known as a social media personality). Some participants explained how social media was considered a means to access their extended family and wider communities.

*Lots of people…go onto WhatsApp and social media to ask family members through different WhatsApp groups about ailments that they might have, or medications that they’re prescribed. (Interview participant 9, SA male, 40)*

First-generation immigrants, tended to use platforms like WhatsApp and Facebook to access health information. Instagram or YouTube videos were accessed directly or shared by personal contacts on WhatsApp – to fill the perceived gaps in official sources of health information (e.g., NHS website), including information on herbal remedies:

‘*On Instagram, I follow herbal doctors – they are online and you can get in touch with them online. (Community setting participant, Black female).*

While community sources were generally thought to be credible, both interview and community event participants acknowledged the variable credibility of online and social media sources. They described several strategies to assess the credibility of online sources of health information other than official websites (e.g., NHS website). One participant, for example, described using online scholarly sources:

*I go to different search engines, because I don’t trust google. I go to google scholar, it gives a lot of information on different topics. (Community setting participant, Black female)*

Other community event participants explained that they assessed expertise based on the number of followers and reviews of the content creators.

*Sometimes on Instagram, you get flashes [by individuals providing information]; I look to see who they follow and who follows them… Also [I consider] how many followers they have. (Community setting participant, Black female.)*

### 3.4. Bridging the credibility gap: Personal and relevant information

The third theme described participants’ preference for health information related to personal experiences and lived experiences and stories shared through online platforms, and for information on familiar, ‘home’ remedies that participants could access and use by themselves, that is, a medicine that was personally known, accessible, and practical. Other participants from the South Asian and Black community prioritised and valued health information that was personal and relevant to them.

#### 3.4.1. Valuing personal experiences and narratives.

Participants frequently sought out the lived experiences of individuals online, including through online discussion groups and the comments section of platforms like YouTube. Some participants regarded personal narratives of people lived experiences as credible and valued sources of information.

*For me, the only way I could determine someone is viable in what they’re saying or believable is if they’ve had it themselves. (Interview participant 14, SA male, 51)*

*I don’t want the academics because [they]…can sometimes be in a different sort of space…I want people that treat patients on a regular basis to then tell me. And then I would want to listen to stroke survivors and their experience of those therapies, and how that’s helped them, and the effects of stroke on the psychological, so the mental, the emotional, the physical aspects of that condition, the implications for work, the implications for your finances, the implications on your life. (Interview participant 5, SA female, 47)*

#### 3.4.2. Desire for familiar, accessible, and practical health information.

There was also a longing for information on treatments that were familiar, accessible, and practical to them. This includes information that may have been in common use in the participant’s family or broader social network, could be acted on by individuals themselves, and/or was directly relevant to their life. This was consistently expressed as a desire for information on diet, nutrition, and non-pharmaceutical remedies, and, frequently, a desire for a more integrative approach, where medical advice from GPs would be complemented by guidance on natural or herbal remedies and nutrition. Both our interviews and community setting observations indicated an appreciation for herbal and ‘home’ remedies, for example:

*There was a time I had toothache, and…I had this idea before that you could put salt in water and use salty water to wash your mouth, and that just really reduced the pain. (Interview participant 26, Black male, 31)*

*We have old remedies. We go to a bush and boil it; it’s from generation to generation. (Community setting participant, Black female).*

The younger participants tended to emphasise nutrition as an alternative or complementary aspect to conventional medical treatments, for example:

*I tend to delve for deeper understanding certain health problems, and which, not treatment medically, but which way of lifestyle is better. Then I look at the research behind the trials... I don’t always think the best treatment is what the NHS offers. (Interview participant 11, SA female, 28)*

*The food that people eat is so vital to actually curing you in some cases. Or even having a detrimental effect on you. So, if that could be blended into the [NHS] website then that would be amazing. (Interview participant 22, SA male, 28)*

Individuals with chronic health conditions also sought a broader perspective on health. They desired guidance on managing the symptoms of their conditions and on living fulfilling lives despite their health condition, bringing a further dimension to the theme ‘personal and relevant’, namely, that of empowerment and control:

*.. and women things that are specific to women…Instead of looking at it from a deficit approach. You need to have an asset-based approach. (Interview participant 21, SA female, 61)*

*People in healthcare talk about managing a condition as opposed to living your life. (Interview participant 5, SA female, 47)*

The absence of advice on sought-after natural remedies impacted the levels of trust in, and expertise ascribed to, health information provided by mainstream healthcare providers because it was considered as relevant and treatment expectations were not fully met, as explained by this participant:

*There are remedies like homeopathy. There is ayurveda. There are many natural remedies and they work. I trust in them, because I have seen for myself, which like years of medication in allopathy they could not cure, homeopathy they did wonder for me. But if you look at the NHS or the doctors, mostly like in the West, they don’t believe homeopathy to be a scientific method. Well, that’s another conflict, you know, so that means half, or even more than half of the information is already missing. (Interview participant 2, SA female, 48)*

Similarly, several participants commented on the absence of nutritional guidance and a focus on prevention. They expressed that they would be more inclined to engage with health information websites if these adopted a proactive, asset-based, and integrated approach to health.

#### 3.4.3. Ethnic-specific health information.

A third aspect of the theme ‘personal and relevant’ revolved around the identity of those seeking health information. A small number of participants, notably those with higher levels of education from both South Asian and Black communities, expressed the importance of statistical information:

*Stats as well, quite important in terms of how common is it? Because if it seems very common, you’ll feel a bit better compared to something which is not as common. (Interview participant 28, Black male, 24)*

But participants, especially those from the Black community, expressed a need for information and data to be ethnicity-specific on how certain ethnicities may be disproportionately affected by specific conditions and how symptoms might present differently.

*They should explain why those kind of ethnic groups are more vulnerable to this kind of disease...It’s the way of eating or it’s because of the weather you came from…It’s not very clear. (Interview participant 1, Black female, 43)*

*For instance…when I’m looking at stuff online…I look at symptoms and sometimes it’s a rash, but I don’t really break out in rashes, so what would that look like on me? (Interview participant 20, Black female, 29*

In general, there was a demand for health information that was perceived as relevant, familiar, accessible, and practical, and that empowered individuals to manage their own health. Absence of this information from mainstream sources of health information, could negatively impact the trust in and perceived expertise of the information source.

## 4. Discussion

The current study sheds light on the impact of perceived credibility (trust and expertise) of health information source preferences in ethnic minority communities and its role in building health literacy. It is important to note that these findings were not homogenous across the different communities included. The study found significant differences in how health information was assessed and trusted. Black communities, with prolonged histories of discrimination and integration, exhibited higher levels of mistrust towards GPs. South Asian participants, who maintain distinct religious and cultural belief systems, placed greater emphasis on the relevance of health information to their cultural practices and beliefs. Issues of intersectionality of education and migration status [32] also need to be taken into account when considering how ethnic communities access and use health information.

Participants’ expectations of the role of health care professionals, particularly the GP and the type of information they sought were shaped by cultural expectations and past experiences in their country of origin, and when these expectations were not met, it impacted their trust in medical advice. Personal contacts within the community, friends, and family were highly trusted and considered experts in health information. Social media platforms were also highly trusted sources of information as they served as an extension of participants’ networks of family and friends. Cultural expectations impacted on how mainstream health information was perceived in terms of source trust and expertise when those expectations were not fulfilled. Our study identified areas of friction (congruence/incongruence), between these expectations and mainstream information sources, such as the perceived absence of personal contact, natural remedy and nutritional information, and ethnically relevant content.

Several behaviours observed in this study, including reliance on social media, family, and friends for health information, are also evident in the general population, particularly among individuals with limited health literacy or limited access to care [4,5]. Prior research has shown that personal relevance, experiential narratives, and perceived trustworthiness shape credibility assessments of online and interpersonal health information sources across diverse populations [7,22]. However, our findings suggest that within the Black and South Asian communities studied, these behaviours are shaped by additional, culturally specific factors. These include historically informed levels of institutional mistrust, expectations formed through prior healthcare systems in countries of origin, and the perceived exclusion from mainstream health messaging. In majority populations, dominant cultural norms are often reflected in official health communications and social networks may more readily diffuse through mainstream social networks. In contrast participants in this study described a perceived misalignment between their own values, experiences, and the information provided through NHS channels. This misalignment appears to intensify reliance on culturally familiar and community-based sources, this distinction underscores the importance of understanding credibility assessments as socially and culturally embedded, rather than solely literacy-driven.

Trust in personal recommendations and relationships was central to determining the credibility of health information sources. The relevance of information to participants’ understanding of health also influenced their assessments of expertise. While trust and expertise evaluations often overlapped, personal sources (such as personal contacts or recommendations) were generally perceived as more trustworthy. Sources that aligned with participants’ own understanding of health (such as ethnically specific or including natural remedies) were viewed as more expert. When mainstream sources lacked credibility in terms of not being personal and relevant, participants turned to alternative sources such as family, community, and non-medical online platforms, creating the potential for misinformation. The current study offers insights on how cultural expectations and experiences influence the assessment of health information credibility and source preferences. It offers an insight into how trust and information relevance shape health-seeking actions and may contribute to building health literacy. The current study makes an important contribution to raise awareness when designing healthcare communications as to why certain messages may be accepted or rejected by diverse communities, thereby improving healthcare accessibility and outcomes. It provides useful guidance for service planners, policy makers and practitioners aiming to create culturally sensitive health information policies and practices that tackle misinformation and promote health equity and literacy.

By highlighting the importance of culturally tailored health information, the findings can guide efforts to enhance patient trust and engagement across diverse populations to address health literacy inequalities [33]. The importance of culturally relevant health communication in promoting health literacy is well recognised [8,34]. Recognising differences in language, social practices, and beliefs allows practitioners to provide more culturally sensitive communications in clinical environments [35] and to promote targeted health interventions [36]. Ethnic representation in messaging increases health knowledge and health information seeking in ethnic communities [37]. Our insights from the current study build on these specific areas of dissemination to identify how differences in community understandings of cultural values, experiences, and expertise may impact information-seeking in ethnic communities. The general importance of trust, personal relevance, and experiential grounding in health information is therefore likely transferable beyond the communities studied. However, the sources through which trust is established and the criteria used to judge relevance appear to vary substantially according to cultural context and social positioning.

We propose the following key findings from our study may be used to build in health literacy in scenarios where there is a risk that health information may be rejected: Firstly, community sources of health information (such as friends, family, religious and community leaders, barbers, shopkeepers with herbal knowledge) were often central to exchanging health information. In addition, for some communities, GPs were considered as trusted and credible sources of health information. However, the lack of time and lack of culturally appropriate information are barriers to accessing this support. Reliance on non-medical and community sources of information can lead to misinformation. Secondly, lived experiences (such as stories shared through online platforms and information on familiar, ‘home’ remedies) shaped perceptions of credibility. Platforms that include lived experiences are prioritised as they are easy to understand, relatable and representative. Thirdly, health information that is compatible and sharable using digital platforms, social media platforms (TikTok, Facebook, Instagram) and sharing platforms such as WhatsApp are preferred as they support informal, community-based information sharing. Fourthly, ethnic communities value and recognise the role of integrating care alongside conventional medical treatments and therapy with guidance on nutrition, herbal remedies, and other culturally significant practices to provide holistic care. Health information that includes these are often seen as credible and culturally relevant. Finally, ethnic-specific information was seen as essential when evaluating whether to trust the information but is often missing from medical sources.

The identification and utilisation of community sources are a key strategy for improving health information uptake, which underscores the importance of adopting culturally sensitive approaches to health communication and resource development [38]. These individuals, including community groups, local GPs, and representative experts, can act as health advocates, intermediaries or knowledge brokers, providing reliable health information and support for ethnically diverse communities [39]. In-person and online narratives of lived experiences hold value as trustworthy illustrations, but online communities and obtaining information from online sources also pose challenges due to misinformation [40,41]. Understanding these factors will shed light on ways to enhance information channels, sources, and formats to encourage health information seeking within ethnic communities and, in turn, improve health disparities [33].

In addition to our own findings, there is evidence that online health information features prominently in ethnic communities [42]. Video platforms could offer valuable opportunities for sharing accurate, vetted health information—especially when addressing the spread of misinformation. Providing culturally relevant information, including ethnically specific statistics and symptom manifestation, is crucial [43]. Including natural alternatives and wellness information may enhance the perceived comprehensiveness of health information. A preventative approach and guidance for living with ailments could support self-management and attract users to health information platforms.

### 4.1. Strengths and limitations of the study

The findings provide insights into the complex system influencing health information source preferences and assessments of credibility by Black and South Asian communities and some strategies to influence them. Robust methods, including using multiple recruitment channels targeting different age groups, and data collection methods (interviews and observational data), were employed, ensuring a comprehensive understanding of the participants’ experiences. The study incorporated input from an advisory group from the community, enhancing the resonance and relevance of the findings. Participant characteristics were diverse, contributing to the richness and depth of insights. The main limitations are that the findings are specific to the studied minority groups and may not be directly transferable to other underserved groups, such as other ethnic minority communities, asylum seekers or undocumented migrants. This is especially the case for the community event where only females were included, limiting the perspectives of men from lower socio-economic backgrounds. At the same time, several underlying processes identified, such as the role of perceived relevance, narrative formats, and trust in interpersonal sources, echo findings from studies of the general population [1,7]. While these processes are shaped by cultural and historical context in this study, they are likely to also overlap with more general patterns in how people assess the credibility of health information. Recognising these overlaps is important, as it helps to distinguish which aspects of credibility assessment are broadly shared and which are shaped by specific social and cultural contexts.

Future research should explore the applicability of the findings to different communities, where there might be cultural reticence in discussing health issues. Further investigation is needed to understand the tension between religious framing and open discussions of health conditions within these communities. Including diverse gender and socio-economic perspectives should be considered in future research. Overall, while the study provides valuable insights into health information access and use, future research should address the limitations by exploring additional underserved groups and including a broader range of perspectives to obtain a comprehensive understanding of the topic. Additionally, in contrast to most studies related to health information seeking and/or health information credibility, this study was conducted in the UK that provides a publicly funded, free at point of use, publicly funded nationalised healthcare system: the National Health Service (NHS).

## 5. Conclusion

The study revealed that cultural expectations and lived experiences significantly influence how different ethnic groups assess the credibility of health information sources. While some credibility-seeking behaviours overlap with those observed in the general population, the unique social, cultural, and historical experiences of these populations shape how and why such behaviours are enacted, resulting in distinct patterns of trust and information use. Health information providers should be aware of the different ways that diverse communities assess credibility in order to impart information effectively and build health literacy. This is especially important with new digital platforms that give rise to more effective health communications at that same time as the potential for misinformation. This paper has offered insights on the risk that health information will be rejected and some key findings that can be inform strategies employed to mitigate the likelihood of rejection. Understanding these insights can enable health professionals create and disseminate personalised, culturally relevant health information content promoting health literacy and health equity.

## Supporting information

S1 File Interview guide. (DOCX)

## Abbreviations

GP: general practitioner
HCP: Healthcare Provider
NHS: National Health Service
CAG: community advisory group
SA: South Asian
UK: United Kingdom
