# Supplementary material for: Understanding interpretations of credibility, health literacy and health information seeking in Black and South Asian ethnic communities in the United Kingdom: A qualitative study
Source: PLoS One. 2026 Sep 2;21(9):e0355294. doi: 10.1371/journal.pone.0355294 (PMC13537547; doi:10.1371/journal.pone.0355294)
Supplement: Supplementary file 1 [file pone.0355294.s001.docx]

**Interview Guide**

**Thank you for agreeing to talk to me. Today, we want to know more about how you find health information. This refers to any information you look for when you decide whether and what action to take related to your health or someone else’s health. There aren’t any right or wrong answers, we are interested in the information that you personally use. Also, please be assured that your information will be kept confidential.**

- Where do you get health information?

How do you go about looking for health information?

- What channels do you use (apps, personal contacts, newspapers/shows)
- What sources (i.e., who do you follow, what websites)?
- Is there anything that might stop you from getting the health information you	need?
- What makes you trust the information you find/receive?
  - How do you normally know if it’s a “good” or “correct” source of information?
  - What information do you not trust?
- Do you ever check where your health information comes from?
  - How do you do this? (or, Why not?)
  - What do you look for? [Answer is likely to include reference to scientific evidence/studies/research if it is relevant to the participant]
- Have you tried to look for scientific evidence/studies/research to make health decisions?
  - Why? Can you give me an example?
  - Where do you go to look for this research?
  - In which situations would you look for research evidence?
  - In which situations do you not look for research evidence? Why?
  - (or Why not?)
  - What do you use instead?
- Would you look for scientific evidence/studies/research to make health decisions?
  - Where would you go to look for this research?
  - Is there anything that makes it difficult for you to look for research?
- What do you like to “see” or “hear” in health information you look for?
  - Statistical information, Personal stories, Summaries of research evidence, Opinions from people you respect
- If you think about how health information is presented, what presentation is most helpful to you?
  - Articles or blogs, Audio (podcasts), Graphic summaries (charts, etc.), Video, In-person or on-line discussions or events, performances or storytelling, other
  - Why do you prefer this presentation?
- Ideally, how would you like to find information that might help you understand your health and treatment more?
  - What would this information look like?
  - How can we make health information more useful to you?

**It was great to talk to you about this – thank you. Do you have any questions for me? Do you want to mention anything which we haven’t covered and which you think might be relevant?**
