# Supplementary material for: Understanding interpretations of credibility, health literacy and health information seeking in Black and South Asian ethnic communities in the United Kingdom: A qualitative study
Source: PLoS One. 2026 Sep 2;21(9):e0355294. doi: 10.1371/journal.pone.0355294 (PMC13537547; doi:10.1371/journal.pone.0355294)
Supplement: Supplementary file 2 [file pone.0355294.s003.docx]

**Rebuttal**

# **Editor comments**

1.Clarification and Integration of Findings: Strengthen the explanation of how the qualitative results specifically reflect the perspectives and characteristics of the two studied ethnic minority groups, especially in relation to your hypothesis. Clarify which aspects of the findings are unique to these groups, and distinguish them from patterns that might also occur in the general population.

*We have added a paragraph on page 5 and page 23/24 which discusses the point. We acknowledge that low literacy may lead to the same reliance on social media, family and friends, but wish to distinguish that the framing of typical health messages may better target and filter into the social networks of majority populations and marginalised communities, such as the ones in the study.*

Provide more explicit connections between the presented findings and the recommendations in the Discussion. Each proposal should be directly supported by, and clearly traceable to, your data. Elaborate on ambiguous terms such as “reliable intermediary,” specifying the expected role, scope, and function based on your study results.

*We have revised the discussion to focus more on key findings of our study rather than prescriptive recommendations. These key findings are more in line with the findings section of the paper (page 25 – 26).*

2.Contextualization for International Readership. Clearly explain the role of the NHS website and GPs as primary sources of “mainstream information” in the UK, as readers outside the UK may be unfamiliar with this context.

*We have added a paragraph on page 4 and 5 explaining the context of UK services in more detail.*

3.Depth of Qualitative Analysis: Refocus the Discussion to emphasize interpretive depth and the richness of the qualitative analysis, rather than predominantly offering prescriptive guidance. This will strengthen the scholarly contribution and theoretical implications of the work.

*The discussion and conclusion have been revised to make them less prescriptive, see pages 24 -27*

4.Methodological Clarity: Include the list or examples of questions used in the semi-structured interviews to improve transparency. Given the qualitative nature of your work, please revise your manuscript to align with the COREQ checklist to ensure completeness and transparency in reporting. Where COREQ items are not applicable, provide a brief explanation. If your methodology extends beyond interviews/focus groups, SRQR may be more appropriate or use Equator website to find appropriate checklist and must attached with appendix.

*We have added the COREQ and a sample interview topic guide to the appendix.*

# **Reviewer comments**

**Reviewer #1:**

**Major points:**

1. The authors hypothesized that ethnic minority groups may differ from the general population in their assessment of health information credibility (P.4, in Introduction). However, it is unclear which parts of the survey results are considered to correspond to the characteristics of these two minority groups. For instance, the people with low literacy in the majority population may also rely on social media, family, or friends rather than the NHS website for information.

*We have added a paragraph on page 5 and 23-24 which clarifies the point. We acknowledge that low literacy may lead to the same reliance on social media, family and friends, but wish to distinguish that the framing of typical health messages may better target and filter into the social networks of majority populations and marginalised communities, such as the one in the study.*

2. Although the health information provided by the NHS and GPs seems to be defined as “mainstream information” throughout the manuscript, readers outside the UK may not realize that the general population commonly uses the NHS website as an initial source of health information. Explaining the social context may help readers understand this.

*We have added a paragraph on page 4, 5 explaining the context of UK services in more detail.*

3. The five proposals in Discussion (p.24) are vague, and the relevance to the results of this study is not clear enough. What does "reliable intermediary" mean in the phrase "utilize reliable intermediaries as effective bridges"? What is the "reliable intermediary” expected to do? Which part of the results led to the suggestion to "use a visually understandable format"? Overall, the new perspectives that the current data brings to the proposal are not clearly described.

*The proposals have been rewritten as key findings see page 25 and 26. The terms ‘reliable intermediary’ has been further explained.*

**Minor points**

1. What kinds of questions were asked during the semi-structured interviews? They need to be provided.

*The topic guide of the interview schedule has been added to the appendix.*

2. Figure 1 is rather confusing. I don't think it's necessary.

*Figure 1 has been removed from appendix and discussion*

**Reviewer #2:**

This paper reports the results of a qualitative study focusing on cultural influences on how Black and South Asian communities in the UK perceive and evaluate the trustworthiness of health information. In addition, the manuscript provides a qualitative analysis of the two ethnic minority groups, yielding valuable insights that integrate social, cultural, and historical perspectives, rather than focusing exclusively on individual experiences. This study addresses an area that is extremely important in health communication research but has not been sufficiently explored to date.

While the inclusion of practical recommendations is valuable, the discussion would benefit from a stronger focus on explicating the qualitative analysis and its interpretive depth, rather than predominantly offering prescriptive guidance.

Several recommendations (e.g., proposals for healthcare providers, policymakers, and digital platforms) are presented in a prescriptive tone.

*The discussion and conclusion have been revised to make them less prescriptive, see pages 24 –27.*
