# Supplementary material for: Understanding interpretations of credibility, health literacy and health information seeking in Black and South Asian ethnic communities in the United Kingdom: A qualitative study
Source: PLoS One. 2026 Sep 2;21(9):e0355294. doi: 10.1371/journal.pone.0355294 (PMC13537547; doi:10.1371/journal.pone.0355294)
Supplement: Supplementary file 3 [file pone.0355294.s004.docx]

Response to reviewers

Editor: "However, one issue remains that should be addressed. As noted by other reviewers, the discussion would benefit from a clearer consideration of whether the observed findings are specific to the study population or may also reflect phenomena present in the general population. A more explicit comparative perspective would strengthen the interpretation of the findings and help clarify the broader applicability and generalizability of the results."

Reviewer #2: "The revised manuscript has been significantly improved in response to the previous reviewers’ comments. The authors have made commendable efforts to address the main concerns, resulting in a clearer structure, improved consistency, and a more comprehensive discussion of the social, cultural, and historical backgrounds of the two ethnic minority groups in particular. However, there are still areas that have not been sufficiently addressed regarding comparisons with phenomena that may also occur in the general population, as pointed out by other reviewers. In the current discussion, it would be beneficial to more clearly examine whether the observed findings are specific to the study population or more broadly applicable. Incorporating such a comparative perspective would further strengthen the interpretation of the results and their generalizability."

*In response to the point raised above, we have made the following revisions:*

- ***In the discussion section, paragraph 3: revised the discussion of comparative findings to be more explicit.*** *Acknowledging more explicitly that several observed behaviours (e.g. reliance on social media, family and friends, preference for experiential information) have also been documented in the general population. This comparison is now explicitly grounded in existing general‑population literature previously discussed (e.g. Berkman et al., 2011; Sbaffi & Rowley, 2017; Hocevar et al., 2017). As well as adding new literature where relevant (e.g Chen et al., 2018)*
- ***In the discussion section, paragraph 3: clarified the distinction between shared behaviours and context‑specific factors,*** *emphasising that while some credibility‑seeking behaviours overlap with those observed in the general population, in Black and South Asian communities these behaviours are shaped by culturally specific factors, including historical experiences of discrimination, expectations formed in countries of origin, and perceived exclusion from mainstream health messaging.*
- ***In the discussion section ,end of paragraph 5: We added a statement clarifying the scope and transferability of the findings.***
- ***Revised the Strengths and Limitations section*** *to more clearly delimit the scope of transferability, acknowledging that findings are context‑specific while noting that some underlying credibility‑assessment processes identified echo those reported in general‑population research, thereby clarifying the intended contribution.*
- ***Refined the Conclusion*** *to reflect this comparative perspective, explicitly stating that although some behaviours overlap with those observed in the general population, the ways they are enacted and interpreted are shaped by distinct cultural and structural experiences, resulting in different patterns of trust and information use.*
- *We have also added a sentence in the abstract to make this point.*
