# Supplementary material for: Understanding interpretations of credibility, health literacy and health information seeking in Black and South Asian ethnic communities in the United Kingdom: A qualitative study
Source: PLoS One. 2026 Sep 2;21(9):e0355294. doi: 10.1371/journal.pone.0355294 (PMC13537547; doi:10.1371/journal.pone.0355294)
Supplement: Supplementary file 4 [file pone.0355294.s005.docx]

Point by Point Response to Reviewers:

**Comments to the Author**

1. If the authors have adequately addressed your comments raised in a previous round of review and you feel that this manuscript is now acceptable for publication, you may indicate that here to bypass the “Comments to the Author” section, enter your conflict of interest statement in the “Confidential to Editor” section, and submit your "Accept" recommendation.

Reviewer #1: All comments have been addressed

Reviewer #2: (No Response)

2. Is the manuscript technically sound, and do the data support the conclusions?

The manuscript must describe a technically sound piece of scientific research with data that supports the conclusions. Experiments must have been conducted rigorously, with appropriate controls, replication, and sample sizes. The conclusions must be drawn appropriately based on the data presented.

Reviewer #1: Yes

Reviewer #2: Yes

3. Has the statistical analysis been performed appropriately and rigorously?

Reviewer #1: Yes

Reviewer #2: Yes

4. Have the authors made all data underlying the findings in their manuscript fully available?

The [PLOS Data policy](http://track.editorialmanager.com/CL0/http:%2F%2Fwww.plosone.org%2Fstatic%2Fpolicies.action%23sharing/1/010f019efc51579c-c81274cc-fd84-4b12-9b13-baa78ffa3e84-000000/cmMphl7XiLUY5sZ-FKcxazerbWj5_P_efHrHYdVfmmU=258) requires authors to make all data underlying the findings described in their manuscript fully available without restriction, with rare exception (please refer to the Data Availability Statement in the manuscript PDF file). The data should be provided as part of the manuscript or its supporting information, or deposited to a public repository. For example, in addition to summary statistics, the data points behind means, medians and variance measures should be available. If there are restrictions on publicly sharing data—e.g. participant privacy or use of data from a third party—those must be specified.

Reviewer #1: Yes

Reviewer #2: Yes

5. Is the manuscript presented in an intelligible fashion and written in standard English?

PLOS ONE does not copyedit accepted manuscripts, so the language in submitted articles must be clear, correct, and unambiguous. Any typographical or grammatical errors should be corrected at revision, so please note any specific errors here.

Reviewer #1: Yes

Reviewer #2: Yes

6. Review Comments to the Author

Please use the space provided to explain your answers to the questions above. You may also include additional comments for the author, including concerns about dual publication, research ethics, or publication ethics. (Please upload your review as an attachment if it exceeds 20,000 characters)

Reviewer #1: (No Response)

Reviewer #2: The revised manuscript has adequately addressed the remaining concern raised in the previous round of review. In particular, the authors have now more clearly distinguished between credibility-seeking behaviours that may also be observed in the general population and those that are shaped by the specific social, cultural, and historical experiences of Black and South Asian communities in the UK. The additions to the Discussion, Strengths and Limitations, Conclusion, and Abstract have strengthened the interpretation of the findings and clarified the intended scope and transferability of the study.
Overall, I find the authors’ response satisfactory. The revised manuscript is clearer, better contextualised, and provides a more balanced account of both shared and population-specific aspects of health information seeking and credibility assessment. I do not think further substantive revision is required.
I recommend acceptance, subject only to minor editorial proofreading. For example, the Abstract appears to contain a typographical error, “United Kingston,” which should be corrected to “United Kingdom.”

Thank you for your help in reviewing and editing, we have gone through the manuscript again and done further proof reading, including the correction to United Kingdom.

7. PLOS authors have the option to publish the peer review history of their article ([what does this mean?](https://track.editorialmanager.com/CL0/https:%2F%2Fjournals.plos.org%2Fplosone%2Fs%2Feditorial-and-peer-review-process%23loc-peer-review-history/1/010f019efc51579c-c81274cc-fd84-4b12-9b13-baa78ffa3e84-000000/suQiBXGfZ9pjNFtxW-hpqrUxY0B7epEWsd1qslOJY_k=258)). If published, this will include your full peer review and any attached files.


If you choose “no”, your identity will remain anonymous but your review may still be made public.


**Do you want your identity to be public for this peer review?** For information about this choice, including consent withdrawal, please see our [Privacy Policy](https://track.editorialmanager.com/CL0/https:%2F%2Fwww.plos.org%2Fprivacy-policy/1/010f019efc51579c-c81274cc-fd84-4b12-9b13-baa78ffa3e84-000000/AJQwVknobcnxiWjBnfsEFkDSqvf3RN_yQ5WQ2PE4CvY=258).

Reviewer #1: No

Reviewer #2: No
