# Supplementary material for: Understanding interpretations of credibility, health literacy and health information seeking in Black and South Asian ethnic communities in the United Kingdom: A qualitative study
Source: PLoS One. 2026 Sep 2;21(9):e0355294. doi: 10.1371/journal.pone.0355294 (PMC13537547; doi:10.1371/journal.pone.0355294)
Supplement: Peer review history

# Peer review: Decision Letter 0

**DOI:** 10.1371/journal.pone.0355294.r001

**Authors:** Muhammad Shahzad Aslam

15 Aug 2025

Dear Dr. Vandrevala,

plosone@plos.org. When you're ready to submit your revision, log on to https://www.editorialmanager.com/pone/ and select the 'Submissions Needing Revision' folder to locate your manuscript file.

- A rebuttal letter that responds to each point raised by the academic editor and reviewer(s). You should upload this letter as a separate file labeled 'Response to Reviewers'.
- A marked-up copy of your manuscript that highlights changes made to the original version. You should upload this as a separate file labeled 'Revised Manuscript with Track Changes'.
- An unmarked version of your revised paper without tracked changes. You should upload this as a separate file labeled 'Manuscript'.

If you would like to make changes to your financial disclosure, please include your updated statement in your cover letter. Guidelines for resubmitting your figure files are available below the reviewer comments at the end of this letter.

If applicable, we recommend that you deposit your laboratory protocols in protocols.io to enhance the reproducibility of your results. Protocols.io assigns your protocol its own identifier (DOI) so that it can be cited independently in the future. For instructions see: https://journals.plos.org/plosone/s/submission-guidelines#loc-laboratory-protocols. Additionally, PLOS ONE offers an option for publishing peer-reviewed Lab Protocol articles, which describe protocols hosted on protocols.io. Read more information on sharing protocols at https://plos.org/protocols?utm_medium=editorial-email&utm_source=authorletters&utm_campaign=protocols.

We look forward to receiving your revised manuscript.

Kind regards,

Muhammad Shahzad Aslam, Ph.D.,M.Phil., Pharm-D

Academic Editor

PLOS ONE

Journal Requirements:

When submitting your revision, we need you to address these additional requirements.

1. Please ensure that your manuscript meets PLOS ONE's style requirements, including those for file naming. The PLOS ONE style templates can be found at

https://journals.plos.org/plosone/s/file?id=wjVg/PLOSOne_formatting_sample_main_body.pdf and

https://journals.plos.org/plosone/s/file?id=ba62/PLOSOne_formatting_sample_title_authors_affiliations.pdf.

2. We note that the grant information you provided in the ‘Funding Information’ and ‘Financial Disclosure’ sections do not match.

When you resubmit, please ensure that you provide the correct grant numbers for the awards you received for your study in the ‘Funding Information’ section.

3.  Thank you for stating the following financial disclosure:

[This article presents independent research funded by the National Institute for Health and Care Research (NIHR). The views expressed are those of the authors and not necessarily those of the NHS, the NIHR or the Department of Health.].

Please state what role the funders took in the study.  If the funders had no role, please state: ""The funders had no role in study design, data collection and analysis, decision to publish, or preparation of the manuscript.""

If this statement is not correct you must amend it as needed.

Please include this amended Role of Funder statement in your cover letter; we will change the online submission form on your behalf.

4. We note that your Data Availability Statement is currently as follows: [All relevant data are within the manuscript and its Supporting Information files.]

Please confirm at this time whether or not your submission contains all raw data required to replicate the results of your study. Authors must share the “minimal data set” for their submission. PLOS defines the minimal data set to consist of the data required to replicate all study findings reported in the article, as well as related metadata and methods (https://journals.plos.org/plosone/s/data-availability#loc-minimal-data-set-definition).

For example, authors should submit the following data:

- The values behind the means, standard deviations and other measures reported;

- The values used to build graphs;

- The points extracted from images for analysis.

Authors do not need to submit their entire data set if only a portion of the data was used in the reported study.

If your submission does not contain these data, please either upload them as Supporting Information files or deposit them to a stable, public repository and provide us with the relevant URLs, DOIs, or accession numbers. For a list of recommended repositories, please see https://journals.plos.org/plosone/s/recommended-repositories.

If there are ethical or legal restrictions on sharing a de-identified data set, please explain them in detail (e.g., data contain potentially sensitive information, data are owned by a third-party organization, etc.) and who has imposed them (e.g., an ethics committee). Please also provide contact information for a data access committee, ethics committee, or other institutional body to which data requests may be sent. If data are owned by a third party, please indicate how others may request data access.

5. Your ethics statement should only appear in the Methods section of your manuscript. If your ethics statement is written in any section besides the Methods, please delete it from any other section.

6. If the reviewer comments include a recommendation to cite specific previously published works, please review and evaluate these publications to determine whether they are relevant and should be cited. There is no requirement to cite these works unless the editor has indicated otherwise.

Additional Editor Comments:

1.Clarification and Integration of Findings:

Strengthen the explanation of how the qualitative results specifically reflect the perspectives and characteristics of the two studied ethnic minority groups, especially in relation to your hypothesis. Clarify which aspects of the findings are unique to these groups, and distinguish them from patterns that might also occur in the general population.

Provide more explicit connections between the presented findings and the recommendations in the Discussion. Each proposal should be directly supported by, and clearly traceable to, your data.

Elaborate on ambiguous terms such as “reliable intermediary,” specifying the expected role, scope, and function based on your study results.

2.Contextualization for International Readership

Clearly explain the role of the NHS website and GPs as primary sources of “mainstream information” in the UK, as readers outside the UK may be unfamiliar with this context.

3..Depth of Qualitative Analysis

Refocus the Discussion to emphasize interpretive depth and the richness of the qualitative analysis, rather than predominantly offering prescriptive guidance. This will strengthen the scholarly contribution and theoretical implications of the work.

4.Methodological Clarity

Include the list or examples of questions used in the semi-structured interviews to improve transparency.

Given the qualitative nature of your work, please revise your manuscript to align with the COREQ checklist to ensure completeness and transparency in reporting. Where COREQ items are not applicable, provide a brief explanation. If your methodology extends beyond interviews/focus groups, SRQR may be more appropriate or use Equator website to find appropriate checklist and must attached with appendix.

[Note: HTML markup is below. Please do not edit.]

Reviewers' comments:

Reviewer's Responses to Questions

**Comments to the Author**

1. Is the manuscript technically sound, and do the data support the conclusions?

Reviewer #1: Yes

Reviewer #2: Yes

**********

2. Has the statistical analysis been performed appropriately and rigorously?

Reviewer #1: N/A

Reviewer #2: Yes

**********

3. Have the authors made all data underlying the findings in their manuscript fully available?

The PLOS Data policy

Reviewer #1: Yes

Reviewer #2: No

**********

4. Is the manuscript presented in an intelligible fashion and written in standard English?

Reviewer #1: Yes

Reviewer #2: Yes

**********

Reviewer #1: This study examined how people from two ethnic minority groups in the UK choose health information sources they rely on. A distinctive feature of this study is that it incorporated the voices of people with limited social connections through observation of a significant number of regional event participants. The study found that past experiences and the discrepancies between cultural expectations and reality prevented them from seeking help from GPs or the NHS website. The lack of ethnic statistics was also identified as a factor undermining trust in the officially provided information. However, the new perspectives provided by the results need to be more clarified.

Major points:

1. The authors hypothesized that ethnic minority groups may differ from the general population in their assessment of health information credibility (P.4, in Introduction). However, it is unclear which parts of the survey results are considered to correspond to the characteristics of these two minority groups. For instance, the people with low literacy in the majority population may also rely on social media, family, or friends rather than the NHS website for information.

2. Although the health information provided by the NHS and GPs seems to be defined as “mainstream information” throughout the manuscript, readers outside the UK may not realize that the general population commonly uses the NHS website as an initial source of health information. Explaining the social context may help readers understand this.

3. The five proposals in Discussion (p.24) are vague, and the relevance to the results of this study is not clear enough. What does "reliable intermediary" mean in the phrase "utilize reliable intermediaries as effective bridges"? What is the "reliable intermediary” expected to do? Which part of the results led to the suggestion to "use a visually understandable format"? Overall, the new perspectives that the current data brings to the proposal are not clearly described.

Minor points

1. What kinds of questions were asked during the semi-structured interviews? They need to be provided.

2. Figure 1 is rather confusing. I don't think it's necessary.

Reviewer #2: This paper reports the results of a qualitative study focusing on cultural influences on how Black and South Asian communities in the UK perceive and evaluate the trustworthiness of health information. In addition, the manuscript provides a qualitative analysis of the two ethnic minority groups, yielding valuable insights that integrate social, cultural, and historical perspectives, rather than focusing exclusively on individual experiences. This study addresses an area that is extremely important in health communication research but has not been sufficiently explored to date.

While the inclusion of practical recommendations is valuable, the discussion would benefit from a stronger focus on explicating the qualitative analysis and its interpretive depth, rather than predominantly offering prescriptive guidance.

Several recommendations (e.g., proposals for healthcare providers, policymakers, and digital platforms) are presented in a prescriptive tone.

**********

what does this mean?). If published, this will include your full peer review and any attached files.

If you choose “no”, your identity will remain anonymous but your review may still be made public.

**Do you want your identity to be public for this peer review?**  For information about this choice, including consent withdrawal, please see our Privacy Policy

Reviewer #1: No

Reviewer #2: No

**********

[NOTE: If reviewer comments were submitted as an attachment file, they will be attached to this email and accessible via the submission site. Please log into your account, locate the manuscript record, and check for the action link "View Attachments". If this link does not appear, there are no attachment files.]

While revising your submission, please upload your figure files to the Preflight Analysis and Conversion Engine (PACE) digital diagnostic tool, https://pacev2.apexcovantage.com/. PACE helps ensure that figures meet PLOS requirements. To use PACE, you must first register as a user. Registration is free. Then, login and navigate to the UPLOAD tab, where you will find detailed instructions on how to use the tool. If you encounter any issues or have any questions when using PACE, please email PLOS at figures@plos.org. Please note that Supporting Information files do not need this step.

# Author comment: Author response to Decision Letter 1

**DOI:** 10.1371/journal.pone.0355294.r002

12 Mar 2026

Rebuttal

Editor comments

1.Clarification and Integration of Findings: Strengthen the explanation of how the qualitative results specifically reflect the perspectives and characteristics of the two studied ethnic minority groups, especially in relation to your hypothesis. Clarify which aspects of the findings are unique to these groups, and distinguish them from patterns that might also occur in the general population.

We have added a paragraph on page 5 and page 23/24 which discusses the point. We acknowledge that low literacy may lead to the same reliance on social media, family and friends, but wish to distinguish that the framing of typical health messages may better target and filter into the social networks of majority populations and marginalised communities, such as the ones in the study.

Provide more explicit connections between the presented findings and the recommendations in the Discussion. Each proposal should be directly supported by, and clearly traceable to, your data. Elaborate on ambiguous terms such as “reliable intermediary,” specifying the expected role, scope, and function based on your study results.

We have revised the discussion to focus more on key findings of our study rather than prescriptive recommendations. These key findings are more in line with the findings section of the paper (page 25 – 26).

2.Contextualization for International Readership. Clearly explain the role of the NHS website and GPs as primary sources of “mainstream information” in the UK, as readers outside the UK may be unfamiliar with this context.

We have added a paragraph on page 4 and 5 explaining the context of UK services in more detail.

3.Depth of Qualitative Analysis: Refocus the Discussion to emphasize interpretive depth and the richness of the qualitative analysis, rather than predominantly offering prescriptive guidance. This will strengthen the scholarly contribution and theoretical implications of the work.

The discussion and conclusion have been revised to make them less prescriptive, see pages 24 -27

4.Methodological Clarity: Include the list or examples of questions used in the semi-structured interviews to improve transparency. Given the qualitative nature of your work, please revise your manuscript to align with the COREQ checklist to ensure completeness and transparency in reporting. Where COREQ items are not applicable, provide a brief explanation. If your methodology extends beyond interviews/focus groups, SRQR may be more appropriate or use Equator website to find appropriate checklist and must attached with appendix.

We have added the COREQ and a sample interview topic guide to the appendix.

Reviewer comments

Reviewer #1:

Major points:

1. The authors hypothesized that ethnic minority groups may differ from the general population in their assessment of health information credibility (P.4, in Introduction). However, it is unclear which parts of the survey results are considered to correspond to the characteristics of these two minority groups. For instance, the people with low literacy in the majority population may also rely on social media, family, or friends rather than the NHS website for information.

We have added a paragraph on page 5 and 23-24 which clarifies the point. We acknowledge that low literacy may lead to the same reliance on social media, family and friends, but wish to distinguish that the framing of typical health messages may better target and filter into the social networks of majority populations and marginalised communities, such as the one in the study.

2. Although the health information provided by the NHS and GPs seems to be defined as “mainstream information” throughout the manuscript, readers outside the UK may not realize that the general population commonly uses the NHS website as an initial source of health information. Explaining the social context may help readers understand this.

We have added a paragraph on page 4, 5 explaining the context of UK services in more detail.

3. The five proposals in Discussion (p.24) are vague, and the relevance to the results of this study is not clear enough. What does "reliable intermediary" mean in the phrase "utilize reliable intermediaries as effective bridges"? What is the "reliable intermediary” expected to do? Which part of the results led to the suggestion to "use a visually understandable format"? Overall, the new perspectives that the current data brings to the proposal are not clearly described.

The proposals have been rewritten as key findings see page 25 and 26. The terms ‘reliable intermediary’ has been further explained.

Minor points

1. What kinds of questions were asked during the semi-structured interviews? They need to be provided.

The topic guide of the interview schedule has been added to the appendix.

2. Figure 1 is rather confusing. I don't think it's necessary.

Figure 1 has been removed from appendix and discussion

Reviewer #2:

This paper reports the results of a qualitative study focusing on cultural influences on how Black and South Asian communities in the UK perceive and evaluate the trustworthiness of health information. In addition, the manuscript provides a qualitative analysis of the two ethnic minority groups, yielding valuable insights that integrate social, cultural, and historical perspectives, rather than focusing exclusively on individual experiences. This study addresses an area that is extremely important in health communication research but has not been sufficiently explored to date.

While the inclusion of practical recommendations is valuable, the discussion would benefit from a stronger focus on explicating the qualitative analysis and its interpretive depth, rather than predominantly offering prescriptive guidance.

Several recommendations (e.g., proposals for healthcare providers, policymakers, and digital platforms) are presented in a prescriptive tone.

The discussion and conclusion have been revised to make them less prescriptive, see pages 24 –27.

Attachment Submitted filename: Response to Reviewers.docx

# Peer review: Decision Letter 1

**DOI:** 10.1371/journal.pone.0355294.r003

**Authors:** Muhammad Shahzad Aslam

31 Mar 2026

Dear Dr. Vandrevala,

Thank you for submitting your manuscript to PLOS ONE. After careful consideration, we feel that it has merit but does not fully meet PLOS ONE’s publication criteria as it currently stands. Therefore, we invite you to submit a revised version of the manuscript that addresses the points raised during the review process.

The revised manuscript has been significantly improved in response to the previous reviewers’ comments. You have made commendable efforts to address the major concerns raised during the earlier round of review. In particular, the manuscript now presents a clearer structure, improved internal consistency, and a more comprehensive discussion of the social, cultural, and historical backgrounds of the two ethnic minority groups examined in the study.

However, one issue remains that should be addressed. As noted by other reviewers, the discussion would benefit from a clearer consideration of whether the observed findings are specific to the study population or may also reflect phenomena present in the general population. A more explicit comparative perspective would strengthen the interpretation of the findings and help clarify the broader applicability and generalizability of the results.

Please submit your revised manuscript by May 15 2026 11:59PM. If you will need more time than this to complete your revisions, please reply to this message or contact the journal office at plosone@plos.org. When you're ready to submit your revision, log on to https://www.editorialmanager.com/pone/ and select the 'Submissions Needing Revision' folder to locate your manuscript file.

- A letter that responds to each point raised by the academic editor and reviewer(s). You should upload this letter as a separate file labeled 'Response to Reviewers'.
- A marked-up copy of your manuscript that highlights changes made to the original version. You should upload this as a separate file labeled 'Revised Manuscript with Track Changes'.
- An unmarked version of your revised paper without tracked changes. You should upload this as a separate file labeled 'Manuscript'.

If you would like to make changes to your financial disclosure, please include your updated statement in your cover letter. Guidelines for resubmitting your figure files are available below the reviewer comments at the end of this letter.

If applicable, we recommend that you deposit your laboratory protocols in protocols.io to enhance the reproducibility of your results. Protocols.io assigns your protocol its own identifier (DOI) so that it can be cited independently in the future. For instructions see: https://journals.plos.org/plosone/s/submission-guidelines#loc-laboratory-protocols. Additionally, PLOS ONE offers an option for publishing peer-reviewed Lab Protocol articles, which describe protocols hosted on protocols.io. Read more information on sharing protocols at https://plos.org/protocols?utm_medium=editorial-email&utm_source=authorletters&utm_campaign=protocols.

As the corresponding author, your ORCID iD is verified in the submission system and will appear in the published article. PLOS supports the use of ORCID, and we encourage all coauthors to register for an ORCID iD and use it as well. Please encourage your coauthors to verify their ORCID iD within the submission system before final acceptance, as unverified ORCID iDs will not appear in the published article. *Only*  the individual author can complete the verification step; PLOS staff *cannot*  verify ORCID iDs on behalf of authors.

We look forward to receiving your revised manuscript.

Kind regards,

Muhammad Shahzad Aslam, Ph.D.,M.Phil., Pharm-D

Academic Editor

PLOS One

**Journal Requirements:**

1. If the reviewer comments include a recommendation to cite specific previously published works, please review and evaluate these publications to determine whether they are relevant and should be cited. There is no requirement to cite these works unless the editor has indicated otherwise.

2. Please review your reference list to ensure that it is complete and correct. If you have cited papers that have been retracted, please include the rationale for doing so in the manuscript text, or remove these references and replace them with relevant current references. Any changes to the reference list should be mentioned in the rebuttal letter that accompanies your revised manuscript. If you need to cite a retracted article, indicate the article’s retracted status in the References list and also include a citation and full reference for the retraction notice.

[Note: HTML markup is below. Please do not edit.]

Reviewers' comments:

Reviewer's Responses to Questions

**Comments to the Author**

Reviewer #1: All comments have been addressed

Reviewer #2: All comments have been addressed

**********

2. Is the manuscript technically sound, and do the data support the conclusions?

Reviewer #1: Yes

Reviewer #2: Yes

**********

3. Has the statistical analysis been performed appropriately and rigorously?

Reviewer #1: N/A

Reviewer #2: N/A

**********

4. Have the authors made all data underlying the findings in their manuscript fully available?

The PLOS Data policy

Reviewer #1: Yes

Reviewer #2: Yes

**********

5. Is the manuscript presented in an intelligible fashion and written in standard English?

Reviewer #1: Yes

Reviewer #2: Yes

**********

Reviewer #1: The revised version focuses solely on the factors impact how two different minority groups evaluate the trustworthiness of medical information. Since the authors did not describe the data based on distance from “mainstream information,” the current explanation of the social context in the UK seems appropriate. The relevance of the new set of proposals to the qualitative data became clearer. The authors responded well to the initial requests.

Reviewer #2: The revised manuscript has been significantly improved in response to the previous reviewers’ comments. The authors have made commendable efforts to address the main concerns, resulting in a clearer structure, improved consistency, and a more comprehensive discussion of the social, cultural, and historical backgrounds of the two ethnic minority groups in particular.

However, there are still areas that have not been sufficiently addressed regarding comparisons with phenomena that may also occur in the general population, as pointed out by other reviewers. In the current discussion, it would be beneficial to more clearly examine whether the observed findings are specific to the study population or more broadly applicable. Incorporating such a comparative perspective would further strengthen the interpretation of the results and their generalizability.

Apart from this point, the manuscript has been appropriately revised and significantly improved. It is considered suitable for publication following minor revisions to address the issues mentioned above.

**********

what does this mean?). If published, this will include your full peer review and any attached files.

If you choose “no”, your identity will remain anonymous but your review may still be made public.

**Do you want your identity to be public for this peer review?**  For information about this choice, including consent withdrawal, please see our Privacy Policy

Reviewer #1: No

Reviewer #2: No

**********

[NOTE: If reviewer comments were submitted as an attachment file, they will be attached to this email and accessible via the submission site. Please log into your account, locate the manuscript record, and check for the action link "View Attachments". If this link does not appear, there are no attachment files.]

To ensure your figures meet our technical requirements, please review our figure guidelines: https://journals.plos.org/plosone/s/figures

You may also use PLOS’s free figure tool, NAAS, to help you prepare publication quality figures: https://journals.plos.org/plosone/s/figures#loc-tools-for-figure-preparation.

NAAS will assess whether your figures meet our technical requirements by comparing each figure against our figure specifications.

# Author comment: Author response to Decision Letter 2

**DOI:** 10.1371/journal.pone.0355294.r004

14 May 2026

In response to the point raised, we have made the following revisions:

• In the discussion section, paragraph 3: revised the discussion of comparative findings to be more explicit. Acknowledging more explicitly that several observed behaviours (e.g. reliance on social media, family and friends, preference for experiential information) have also been documented in the general population. This comparison is now explicitly grounded in existing general population literature previously discussed (e.g. Berkman et al., 2011; Sbaffi & Rowley, 2017; Hocevar et al., 2017). As well as adding new literature where relevant (e.g Chen et al., 2018)

• In the discussion section, paragraph 3: clarified the distinction between shared behaviours and context specific factors, emphasising that while some credibility seeking behaviours overlap with those observed in the general population, in Black and South Asian communities these behaviours are shaped by culturally specific factors, including historical experiences of discrimination, expectations formed in countries of origin, and perceived exclusion from mainstream health messaging.

• In the discussion section ,end of paragraph 5: We added a statement clarifying the scope and transferability of the findings.

• Revised the Strengths and Limitations section to more clearly delimit the scope of transferability, acknowledging that findings are context specific while noting that some underlying credibility assessment processes identified echo those reported in general population research, thereby clarifying the intended contribution.

• Refined the Conclusion to reflect this comparative perspective, explicitly stating that although some behaviours overlap with those observed in the general population, the ways they are enacted and interpreted are shaped by distinct cultural and structural experiences, resulting in different patterns of trust and information use.

• We have also added a sentence in the abstract to make this point.

Attachment Submitted filename: Responses to reviewers.docx

# Peer review: Decision Letter 2

**DOI:** 10.1371/journal.pone.0355294.r005

**Authors:** Indra Kiling

24 Jun 2026

Dear Dr. Vandrevala,

Thank you for submitting your manuscript to PLOS ONE. After careful consideration, we feel that it has merit but does not fully meet PLOS ONE’s publication criteria as it currently stands. Therefore, we invite you to submit a revised version of the manuscript that addresses the points raised during the review process.

Please submit your revised manuscript by Aug 08 2026 11:59PM. If you will need more time than this to complete your revisions, please reply to this message or contact the journal office at plosone@plos.org. When you're ready to submit your revision, log on to https://www.editorialmanager.com/pone/ and select the 'Submissions Needing Revision' folder to locate your manuscript file.

- A letter that responds to each point raised by the academic editor and reviewer(s). You should upload this letter as a separate file labeled 'Response to Reviewers'.
- A marked-up copy of your manuscript that highlights changes made to the original version. You should upload this as a separate file labeled 'Revised Manuscript with Track Changes'.
- An unmarked version of your revised paper without tracked changes. You should upload this as a separate file labeled 'Manuscript'.

If you would like to make changes to your financial disclosure, please include your updated statement in your cover letter. Guidelines for resubmitting your figure files are available below the reviewer comments at the end of this letter.

If applicable, we recommend that you deposit your laboratory protocols in protocols.io to enhance the reproducibility of your results. Protocols.io assigns your protocol its own identifier (DOI) so that it can be cited independently in the future. For instructions see: https://journals.plos.org/plosone/s/submission-guidelines#loc-laboratory-protocols. Additionally, PLOS ONE offers an option for publishing peer-reviewed Lab Protocol articles, which describe protocols hosted on protocols.io. Read more information on sharing protocols at https://plos.org/protocols?utm_medium=editorial-email&utm_source=authorletters&utm_campaign=protocols.

As the corresponding author, your ORCID iD is verified in the submission system and will appear in the published article. PLOS supports the use of ORCID, and we encourage all coauthors to register for an ORCID iD and use it as well. Please encourage your coauthors to verify their ORCID iD within the submission system before final acceptance, as unverified ORCID iDs will not appear in the published article. *Only*  the individual author can complete the verification step; PLOS staff *cannot*  verify ORCID iDs on behalf of authors.

We look forward to receiving your revised manuscript.

Kind regards,

Indra Yohanes Kiling, Ph.D

Academic Editor

PLOS One

Journal Requirements:

1. If the reviewer comments include a recommendation to cite specific previously published works, please review and evaluate these publications to determine whether they are relevant and should be cited. There is no requirement to cite these works unless the editor has indicated otherwise.

2. Please review your reference list to ensure that it is complete and correct. If you have cited papers that have been retracted, please include the rationale for doing so in the manuscript text, or remove these references and replace them with relevant current references. Any changes to the reference list should be mentioned in the rebuttal letter that accompanies your revised manuscript. If you need to cite a retracted article, indicate the article’s retracted status in the References list and also include a citation and full reference for the retraction notice.

[Note: HTML markup is below. Please do not edit.]

Reviewers' comments:

Reviewer's Responses to Questions

**Comments to the Author**

Reviewer #1: All comments have been addressed

Reviewer #2: (No Response)

**********

2. Is the manuscript technically sound, and do the data support the conclusions?

Reviewer #1: Yes

Reviewer #2: Yes

**********

3. Has the statistical analysis been performed appropriately and rigorously?

Reviewer #1: Yes

Reviewer #2: Yes

**********

4. Have the authors made all data underlying the findings in their manuscript fully available?

The PLOS Data policy

Reviewer #1: Yes

Reviewer #2: Yes

**********

5. Is the manuscript presented in an intelligible fashion and written in standard English?

Reviewer #1: Yes

Reviewer #2: Yes

**********

Reviewer #1: (No Response)

Reviewer #2: The revised manuscript has adequately addressed the remaining concern raised in the previous round of review. In particular, the authors have now more clearly distinguished between credibility-seeking behaviours that may also be observed in the general population and those that are shaped by the specific social, cultural, and historical experiences of Black and South Asian communities in the UK. The additions to the Discussion, Strengths and Limitations, Conclusion, and Abstract have strengthened the interpretation of the findings and clarified the intended scope and transferability of the study.

Overall, I find the authors’ response satisfactory. The revised manuscript is clearer, better contextualised, and provides a more balanced account of both shared and population-specific aspects of health information seeking and credibility assessment. I do not think further substantive revision is required.

I recommend acceptance, subject only to minor editorial proofreading. For example, the Abstract appears to contain a typographical error, “United Kingston,” which should be corrected to “United Kingdom.”

**********

what does this mean?). If published, this will include your full peer review and any attached files.

If you choose “no”, your identity will remain anonymous but your review may still be made public.

**Do you want your identity to be public for this peer review?**  For information about this choice, including consent withdrawal, please see our Privacy Policy

Reviewer #1: No

Reviewer #2: No

**********

[NOTE: If reviewer comments were submitted as an attachment file, they will be attached to this email and accessible via the submission site. Please log into your account, locate the manuscript record, and check for the action link "View Attachments". If this link does not appear, there are no attachment files.]

To ensure your figures meet our technical requirements, please review our figure guidelines: https://journals.plos.org/plosone/s/figures

You may also use PLOS’s free figure tool, NAAS, to help you prepare publication quality figures: https://journals.plos.org/plosone/s/figures#loc-tools-for-figure-preparation.

NAAS will assess whether your figures meet our technical requirements by comparing each figure against our figure specifications.

# Author comment: Author response to Decision Letter 3

**DOI:** 10.1371/journal.pone.0355294.r006

3 Jul 2026

"Reviewer #2: The revised manuscript has adequately addressed the remaining concern raised in the previous round of review. In particular, the authors have now more clearly distinguished between credibility-seeking behaviours that may also be observed in the general population and those that are shaped by the specific social, cultural, and historical experiences of Black and South Asian communities in the UK. The additions to the Discussion, Strengths and Limitations, Conclusion, and Abstract have strengthened the interpretation of the findings and clarified the intended scope and transferability of the study.

Overall, I find the authors’ response satisfactory. The revised manuscript is clearer, better contextualised, and provides a more balanced account of both shared and population-specific aspects of health information seeking and credibility assessment. I do not think further substantive revision is required.

I recommend acceptance, subject only to minor editorial proofreading. For example, the Abstract appears to contain a typographical error, “United Kingston,” which should be corrected to “United Kingdom.”"

Thank you for your help in reviewing and editing, we have gone through the manuscript again and done further proof reading, including the correction to United Kingdom.

Attachment Submitted filename: Response_to_reviewers_auresp_3.docx

# Editor report: Decision Letter 3

**DOI:** 10.1371/journal.pone.0355294.r007

**Authors:** Indra Kiling

21 Jul 2026

Understanding interpretations of credibility, health literacy and health information seeking in Black and South Asian ethnic communities in the United Kingdom: A qualitative study

PONE-D-25-30785R3

Dear Dr. Vandrevala,

We’re pleased to inform you that your manuscript has been judged scientifically suitable for publication and will be formally accepted for publication once it meets all outstanding technical requirements.

Within one week, you’ll receive an e-mail detailing the required amendments. When these have been addressed, you’ll receive a formal acceptance letter and your manuscript will be scheduled for publication.

An invoice will be generated when your article is formally accepted. Please note, if your institution has a publishing partnership with PLOS and your article meets the relevant criteria, all or part of your publication costs will be covered. Please make sure your user information is up-to-date by logging into Editorial Manager at Editorial Manager® and clicking the ‘Update My Information' link at the top of the page. For questions related to billing, please contact billing support.

If your institution or institutions have a press office, please notify them about your upcoming paper to help maximize its impact. If they’ll be preparing press materials, please inform our press team as soon as possible -- no later than 48 hours after receiving the formal acceptance. Your manuscript will remain under strict press embargo until 2 pm Eastern Time on the date of publication. For more information, please contact onepress@plos.org.

Kind regards,

Indra Yohanes Kiling, Ph.D

Academic Editor

PLOS One

Additional Editor Comments (optional):

Reviewers' comments:

# Editor report: Acceptance letter

**DOI:** 10.1371/journal.pone.0355294.r008

**Authors:** Indra Kiling

PONE-D-25-30785R3

PLOS One

Dear Dr. Vandrevala,

I'm pleased to inform you that your manuscript has been deemed suitable for publication in PLOS One. Congratulations! Your manuscript is now being handed over to our production team.

At this stage, our production department will prepare your paper for publication. This includes ensuring the following:

* All references, tables, and figures are properly cited

* All relevant supporting information is included in the manuscript submission,

* There are no issues that prevent the paper from being properly typeset

You will receive further instructions from the production team, including instructions on how to review your proof when it is ready. Please keep in mind that we are working through a large volume of accepted articles, so please give us a few days to review your paper and let you know the next and final steps.

Lastly, if your institution or institutions have a press office, please let them know about your upcoming paper now to help maximize its impact. If they'll be preparing press materials, please inform our press team within the next 48 hours. Your manuscript will remain under strict press embargo until 2 pm Eastern Time on the date of publication. For more information, please contact onepress@plos.org.

You will receive an invoice from PLOS for your publication fee after your manuscript has reached the completed accept phase. If you receive an email requesting payment before acceptance or for any other service, this may be a phishing scheme. Learn how to identify phishing emails and protect your accounts at https://explore.plos.org/phishing.

If we can help with anything else, please email us at customercare@plos.org.

Thank you for submitting your work to PLOS One and supporting open access.

Kind regards,

PLOS ONE Editorial Office Staff

on behalf of

Dr. Indra Yohanes Kiling

Academic Editor

PLOS One
